# The histone deacetylase SIRT6, a critical modulator of metabolism and tumorigenesis

**DOI:** 10.1186/1753-6561-6-S3-O17

**Published:** 2012-06-01

**Authors:** Carlos Sebastian, Lei Zhong, Magali Silberman, Deborah Toiber, Barbara Martinez, Jean-Pierre Etchegaray, Claudia Cosentino, Sofia Giacosa, Raul Mostoslavsky

**Affiliations:** 1 The Massachusetts General Hospital Cancer Center, Harvard Medical School, Boston, MA USA

## 

Efficient glucose metabolism is critical for maintaining cellular viability. Under normal nutrient and oxygen conditions, glucose is converted to pyruvate, entering the mitochondria for oxidative phosphorylation and ATP production. Under hypoxia or nutrient stress, metabolism is switched to glycolysis, increasing lactate production and reducing mitochondrial respiration, a switch known to play an important role in cancer cells, as defined by Otto Warburg decades ago. Little is known whether chromatin plays a role in carbohydrate flux. The yeast Sir2 protein is an NAD-dependent histone deacetylase that senses the metabolic status of the cell and functions as a chromatin silencer to promote lifespan and genomic stability. Recently, we discovered that the mammalian SIRT6 is a chromatin factor that influences glucose metabolism and DNA repair. In mice, SIRT6-deficiency provokes a profound and lethal hypoglycemia which culminates in accelerated death. At the cellular level, SIRT6 inactivation leads to increased cellular glucose uptake, higher lactate production and decreased mitochondrial activity. Our results indicate that SIRT6 directly regulates expression of several key glycolytic genes. In this context, SIRT6 co-represses Hif1α, acting as a histone H3 lysine9 (H3K9) deacetylase to inhibit expression of glycolytic genes (Zhong et al, 2010). Strikingly, our new studies indicate that the “glycolytic switch” observed in the absence of SIRT6 provides a unique growth advantage in the context of tumorigenesis. Indeed, we find that SIRT6 deficiency causes transformation even in the absence of oncogenic signaling, and SIRT6 levels determine progression and disease-free survival in various human cancers. Our results suggest that SIRT6 might play a critical role in modulating the Warburg effect.

## References

[B1] Zhong L D’Urso A Toiber D Sebastian C Henry RE Vadysirisack DD Guimaraes A Marinelli B Wikstrom JD Nir T Clish CB Vaitheesvaran Iliopoulos OB Kurland I Dor Y Weissleder R Shirihai OS Ellisen L Espinosa JM Mostoslavsky R The histone deacetylase SIRT6 regulates glucose homeostasis via Hif1a Cell 2010 140 280 293 20141841 10.1016/j.cell.2009.12.041 PMC2821045

